# Mammographic density and breast cancer risk: a mediation analysis

**DOI:** 10.1186/s13058-016-0750-0

**Published:** 2016-09-21

**Authors:** Megan S. Rice, Kimberly A. Bertrand, Tyler J. VanderWeele, Bernard A. Rosner, Xiaomei Liao, Hans-Olov Adami, Rulla M. Tamimi

**Affiliations:** 1 Clinical and Translational Epidemiology Unit, Department of Medicine, Massachusetts General Hospital, 55 Fruit Street, Bartlett 9, Boston, MA 02116 USA; 2 Slone Epidemiology Center, Boston University, Boston, MA USA; 3 Department of Epidemiology, Harvard T.H. Chan School of Public Health, Boston, MA USA; 4 Channing Division of Network Medicine, Brigham and Women’s Hospital/Harvard Medical School, Boston, MA USA; 5 Department of Biostatistics, Harvard T. H. Chan School of Public Health, Boston, MA USA; 6 Department of Epidemiology, Institute of Health and Society, University of Oslo, Oslo, Norway; 7 Department of Medical Epidemiology and Biostatistics, Karolinska Institute, Stockholm, Sweden

## Abstract

**Background:**

High mammographic density (MD) is a strong risk factor for breast cancer. However, it is unclear whether high MD is an intermediate phenotype or whether breast cancer risk factors influence breast cancer risk and MD independently.

**Methods:**

Our study population included 1290 invasive breast cancer cases and 3422 controls from the Nurses’ Health Studies. We estimated the percent of the total association between the risk factor and breast cancer that was mediated by MD.

**Results:**

In both pre- and postmenopausal women, the association between history of biopsy-confirmed benign breast disease and risk was partially mediated by percent MD (percent mediated (PM) = 17 %, *p* < 0.01 and PM = 33 %, *p* = 0.04, respectively). In premenopausal women, the associations between early life body size (adolescent somatotype and BMI at age 18) and breast cancer risk were substantially mediated by percent MD (PM = 73 %, *p* = 0.05 and PM = 82 %, *p* = 0.04, respectively). In postmenopausal women, the proportion of the associations of childhood somatotype and adolescent somatotype that were mediated by percent MD were lower (PM = 26 %, *p* = 0.01 for both measures). Hormone therapy use at mammogram was significantly mediated by percent MD in postmenopausal women (PM = 22 %, *p* < 0.01). Associations with other risk factors, such as age at menarche or family history of breast cancer, were not mediated by percent MD.

**Conclusions:**

Percent MD partially mediated some of the associations between risk factors and breast cancer, though the magnitude varied by risk factor and menopausal status. These findings suggest that high MD may be an intermediate in some biological pathways for breast cancer development.

**Electronic supplementary material:**

The online version of this article (doi:10.1186/s13058-016-0750-0) contains supplementary material, which is available to authorized users.

## Background

Mammographic density (MD), or the radiographic appearance of the breast on a mammogram, is a strong risk factor for breast cancer [1]. Dense breast tissue appears light on a mammogram and is comprised of epithelial and stromal tissue whereas non-dense tissue, comprised of fat, appears dark. Women with over 75 % dense tissue have four to six times the risk of breast cancer compared to those with very little to no dense tissue [2]. Further, a number of reproductive and lifestyle risk factors for breast cancer have been consistently associated with MD [3–8]. Since MD is associated with both lifestyle and reproductive risk factors and with risk of breast cancer, it has been hypothesized that MD may be an intermediate marker of breast cancer risk [9]. As a result, percent MD has been proposed as a potential surrogate marker for breast cancer risk in intervention trials [8]. However, it is unknown the extent to which reproductive and lifestyle factors influence breast cancer risk through their effects on MD and the extent to which they influence risk though other pathways,. While some prior studies have examined whether MD mediates the associations with breast cancer risk for some risk factors, such as body size and hormone therapy use, most studies have not attempted to quantify the extent to which the associations are mediated by MD [10, 11]. For example, in a prior analysis in the Nurses’ Health Study (NHS) and NHSII, we observed that the associations between early life body size (e.g., body mass index [BMI] at age 18) and breast cancer risk were attenuated after adjustment for percent MD, suggesting that MD at least partially mediated the associations [10]. However, we did not estimate the extent to which MD mediated the associations nor did we examine other established or probable breast cancer risk factors, such as family history of breast cancer or reproductive factors [10]. Quantifying the extent to which MD mediates the associations with established risk factors would provide insight into breast cancer etiology, including the role of MD in breast cancer risk. Therefore, the purpose of this analysis was to quantify the extent to which the associations between established lifestyle and reproductive breast cancer risk factors and breast cancer risk are mediated by MD in a large sample of pre- and postmenopausal women in the NHS and NHSII.

## Methods

### Study population

In 1976, 121,700 female registered nurses aged 30 to 55 from 11 US states completed an initial questionnaire forming the NHS cohort. The NHSII began in 1989 when 116,430 female registered nurses, aged 25 to 42, from 14 US states completed a baseline questionnaire. Both cohorts are followed by biennially mailed questionnaires to collect information on exposures and covariates as well as incident diseases. In 1989–1990, we obtained blood samples from 32,826 NHS participants, ages 43 to 70. From 1996 to 1999, 29,611 NHSII members, aged 32 to 45 years, provided blood samples. Our mammogram collection was conducted within the case-control studies of breast cancer nested in the NHS/NHSII blood subcohorts, which have been described previously [12–14]. Briefly, cases were identified on biennial questionnaires or through death records. These participants or their next of kin were asked for permission to obtain their medical records, which were reviewed by study investigators. As we confirm 99 % of reported cases of breast cancer for whom we are able to obtain medical records, all diagnoses of breast cancer confirmed by the participant or medical records are included as cases in the nested case-control studies. One or two controls were matched to breast cancer cases on age, menopausal status at blood draw and diagnosis, current hormone therapy (HT) use, month, time of day, fasting status at time of blood collection, and luteal day (NHSII timed samples only). Mammograms conducted as close as possible to the date of blood draw were collected for cases and matched controls diagnosed after blood collection, but before June 1, 2004 (NHS) or June 1, 2007 (NHSII). Further, we collected additional mammograms conducted around 1997 from NHSII breast cancer cases and controls who participated in the NHSII cheek cell collection. In total, mammograms were collected from 2062 breast cancer cases and 4196 matched controls. We further excluded women with missing data on putative MD and breast cancer risk factors, specifically menopausal status (*n* = 385), current body mass index (BMI) (*n* = 112), BMI at age 18 (*n* = 184), parity (*n* = 45), age at first birth (*N* = 4), age at menarche (*n* = 23), adolescent somatotype (*n* = 173), and hormone therapy (HT) use (*n* = 194, postmenopausal only). Next, we excluded women with outlying values based on the generalized extreme studentized deviate many-outlier detection approach [15] for the following variables: BMI (*n* = 16), BMI at age 18 (*n* = 18), age at first birth (*n* = 8), and age at menarche (*n* = 2). The study was approved by the Committee on the Use of Human Subjects in Research at the Brigham and Women’s Hospital.

### Mammographic density

A Lumysis 85 laser film scanner (Lumisys, Sunnyvale, CA, USA) was used to digitize the craniocaudal views of both breasts for all mammograms in the NHS and for the first two batches of mammograms in the NHSII. The third batch of mammograms in the NHSII was scanned using a VIDAR CAD PRO Advantage scanner (VIDAR Systems Corporation; Herndon, VA, USA) using comparable resolution of 150 dots per inch and 12 bit depth. We measured absolute dense area, absolute non-dense area (the total area minus the dense area), and percent MD (the dense area divided by the total area) using the Cumulus software for computer-assisted thresholding (Canto Software, San Francisco, CA, USA). Next, we averaged the density parameters of both breasts. To assess the potential variability in percent MD by scanner, we conducted a pilot study of 50 mammograms. These mammograms were scanned using both the Lumysis 85 laser scanner and the VIDAR CAD PRO Advantage scanner; percent MD was measured by the same observer using Cumulus. The correlation between percent density as measured by the two scanners was 0.88. Two observers read the mammograms from NHS participants in two batches. In NHSII, a single observer read the mammograms in three batches (batches 1 and 2 were read 3 years apart, batches 2 and 3 were read 3 years apart). A small number of mammograms (*N* = 50) were included in all three NHSII batches. While there was high reproducibility within each batch, there was evidence of between-batch variability in the NHSII. Therefore, for the overall NHSII breast cancer case-control mammography dataset, we used multivariable linear regression models to estimate the effect of batch on density measurements, controlling for age, menopausal status, BMI, and case-control status [16, 17]. We then adjusted density measurements in the second and third NHSII batches by adding the coefficient for each mammogram batch to the raw value to estimate the measurements that would have been obtained if the mammogram had been included in the first batch. For all batches, readers were blinded to case-control status.

### Selected breast cancer risk factors

NHS/NHSII participants reported their height and age at menarche on the baseline questionnaires. Personal history of benign breast disease (BBD), including whether it was confirmed by biopsy, and weight were queried on the baseline and all biennial questionnaires. Weight at age 18 was asked in 1980 (NHS) and in 1989 (NHSII). Current BMI and BMI at age 18 were calculated as weight(kg)/height(m)^2^. In 1988 (NHS) and 1989 (NHSII), participants were asked to report their body size at ages 5, 10, and 20 using a 9-level figure with a value of 1 being the leanest figure and 9 being the heaviest figure [10]. Childhood somatotype was calculated as the average of the somatotypes at ages 5 and 10; adolescent somatotype was calculated as the average of somatotypes at ages 10 and 20. Participants reported whether they had a first-degree relative (mother or sister) with a diagnosis of breast cancer at baseline, in 1982 (NHS), and then every 4 years beginning in 1988 (NHS) or 1997 (NHSII). In the NHS, parity and age at first birth were queried on the baseline questionnaire as well as biennial questionnaires until 1984 and again in 1996 in order to update the data on each participant’s lifetime pregnancy history. In the NHSII, parity and age at first birth were asked on the baseline questionnaire and respondents reported parity, including year of each pregnancy, on each subsequent biennial questionnaire. Birth index (a measure which incorporates the number of births, age at each birth, and time since each birth) was calculated as the sum of the total years from each birth to a woman’s age at mammogram (or age at menopause if postmenopausal) with nulliparous women receiving a value of 0 [18]. Breastfeeding history was asked in 1986 in the NHS and in 1993 and 1997 in the NHSII. In the NHS, alcohol intake was first asked in 1980 and subsequently in 1984, 1986, and every 4 years afterwards. In the NHSII, alcohol intake was asked at baseline, in 1991, and every 4 years afterwards. In the NHS and NHSII, women were asked about their hormone therapy use on the baseline and biennial questionnaires. For all variables included on multiple biannual questionnaires, we used information from the most recent questionnaire prior to the date of the mammogram.

### Statistical analysis

For all analyses, percent MD, dense area, and non-dense area were square-root transformed to improve normality. We used linear regression to estimate the differences in percent MD, dense area, and non-dense area by reproductive and lifestyle risk factors among the controls. To assess the extent to which the associations between each of the selected exposures and breast cancer risk are mediated by MD, we used the method for mediation analysis outlined by Lin et al. implemented using the SAS macro developed by Spiegelman and colleagues at the Harvard T.H. Chan School of Public Health [19]. Briefly, this method uses data augmentation and logistic regression to compare a model unadjusted for the hypothesized mediator to a model adjusted for the hypothesized mediator. Additional information on this method and the macro can be found at: http://www.hsph.harvard.edu/donna-spiegelman/software/mediate/. Using this macro, we estimated the odds ratio (OR) and 95 % confidence interval (CI) for (a) the association between the selected exposure and breast cancer risk not adjusted for MD (i.e., the total association between the exposure and breast cancer risk) and (b) the association between the selected exposure and breast cancer risk adjusted for MD (i.e., the association between the exposure and breast cancer risk not through MD). We also estimated the percent of the total association (on the log odds scale) between the exposure and breast cancer risk that was mediated by MD using the following equation: 1-(lnOR_adjusted_/lnOR_unadjusted_). Our primary analysis examined percent MD as a potential mediator since percent MD is a stronger predictor of subsequent breast cancer risk than dense or non-dense area [20]. However, as both dense area and non-dense area have been independently associated with breast cancer risk in the NHS/NHSII, we also examined mediation of the associations by these measures in secondary analyses [20]. Further, in secondary analyses, we estimated the percent mediated by percent MD for each of the selected exposures using an alternative method outlined by VanderWeele and Vansteelandt [21], which, in contrast to the Lin method, also models interaction between the exposure and the mediator. Using this method, we calculated ORs and 95 % CIs for (a) the natural direct effect (NDE) (i.e., the effect of the exposure on breast cancer risk not through percent MD if percent MD was fixed at the level that it would be if the exposure was set to the referent category), (b) the natural indirect effect (NIE) (i.e., the effect of the exposure on breast cancer risk through percent MD), and (c) the total association between the exposure and breast cancer risk. In the presence of interaction between the exposure and percent MD, the NDE will vary depending on the referent category of the exposure (since percent MD is fixed at the level that it would be if the exposure was set to the referent category). Therefore, for all exposures we present the mediation analysis for both contrasts (e.g., for family history of breast cancer compared to no family history of breast cancer as well as for no family history of breast cancer compared to family history of breast cancer). In the absence of interaction between the exposure and percent MD, the OR_NDE_ calculated using the VanderWeele and Vansteelandt method will not vary by the referent category and will approximate the OR_adjusted_ calculated using the Lin method. For the VanderWeele and Vansteelandt method, we also computed the percent of the total association (on the log odds scale) that was mediated by MD using the formula 1-(lnOR_NDE_/lnOR_total_). We further used sensitivity analyses to assess how robust evidence for mediation was to potential unmeasured common causes of MD and breast cancer risk [9, 22]. Lastly, we evaluated whether there was statistically significant interaction between each exposure and percent MD by including an interaction term between the continuous exposure and continuous percent MD and using the Wald test to assess the significance. As we did not observe significant interaction with percent MD for most of the exposures, the Lin method is presented as the primary analysis.

Exposure variables were modeled continuously except for the following binary variables: history of BBD, nulliparity, family history of breast cancer, past HT use (vs never), and current HT use (vs never). Total months breastfeeding was categorized as: <1, 1–3, 4–6, 7–11, 12–17, 18–23, 24–35, and 36+. We assigned women the value of the midpoint of their category (or 36 for the highest category) and modeled the category medians continuously. All models were adjusted for matching factors, specifically age (continuous), time of blood collection (12 am–5:59 am, 6:00 am–7:59 am, 8:00 am–11:59 pm), cohort/batch (NHS batch 1, NHS batch 2, NHSII), fasting status (no, yes), and HT use (never, past, current; postmenopausal only). Models were also adjusted for potential confounders of the association between the exposures and breast cancer risk, potential confounders of the association between the exposures and MD, and potential confounders of the association between MD and breast cancer risk. These covariates were current BMI (continuous), BMI at age 18 (continuous), adolescent somatotype (continuous), history of BBD (no, yes), nulliparity (no, yes), parity (continuous), age at first birth (continuous), and age at menarche (continuous). In our primary analysis, early life somatotypes and BMI at age 18 were not adjusted for adolescent somatotype, BMI at age 18, or current BMI as these variables are likely on the causal pathway. However, we adjusted early life somatotypes and BMI at age 18 by current BMI in sensitivity analyses. Further, weight change since age 18 was not adjusted for current BMI. All continuous variables, including MD measures, were centered at the median. As the associations between some exposures and breast cancer risk vary by menopausal status and percent MD is lower in postmenopausal women compared to premenopausal women, all analyses were conducted separately in pre- and postmenopausal women (menopausal status was defined at the time of the mammogram). Analyses were conducted using SAS version 9.4 (SAS Institute, Cary, NC, USA) and results were considered statistically significant if *p* ≤ 0.05.

## Results

Table 1 presents participant characteristics at the NHS/NHSII questionnaire cycle prior to the date of the mammogram by case-control and menopausal status. Participant characteristics among the controls by quartile of percent MD and menopausal status are presented in Additional file 1: Table S1. Further, the associations between each of the exposures and MD measures among the controls, adjusting for potential confounders, are presented in Additional file 2: Table S2. The average time between mammogram and breast cancer diagnosis was 4.7 years (standard deviation [SD] = 3.4) in women premenopausal at the time of the mammogram and 4.3 years (SD = 3.5) in postmenopausal women. Among cases who were premenopausal at mammogram, the mean age at diagnosis was 50.9; while the mean age at diagnosis among cases who were postmenopausal at mammogram was 64.4. Of the three measures, percent MD was most strongly associated with breast cancer risk, adjusting for age and potential confounders in pre- and postmenopausal women (OR per standard deviation increase = 1.51, 95 % CI: 1.33, 1.71 and OR per standard deviation increase = 1.34, 95 % CI: 1.21, 1.50; respectively). In both pre- and postmenopausal women, dense area was positively associated with breast cancer risk (OR per standard deviation increase = 1.48, 95 % CI: 1.32, 1.65 and OR per standard deviation increase = 1.26, 95 % CI 1.13, 1.41; respectively) whereas non-dense area was inversely associated with risk (OR per standard deviation increase = 0.80, 95 % CI: 0.69, 0.93 and OR per standard deviation increase = 0.81, 95 % CI: 0.71, 0.94; respectively).

**Table 1:** Selected risk factors at time of mammography by case-control status and menopausal status at the time of the mammogram (NHS/NHSII)

	Premenopausal		Postmenopausal	
	Cases *N* = 559	Controls *N* = 1727	Cases *N* = 731	Controls *N* = 1695
Mean (SD)				
Age^a^	46.2 (4.6)	46.1 (4.3)	60.1 (7.0)	59.6 (7.4)
Current BMI (kg/m^2^)	25.2 (4.8)	25.4 (5.1)	25.9 (4.6)	26.1 (5.0)
Childhood somatotype	2.5 (1.2)	2.6 (1.2)	2.2 (1.2)	2.4 (1.3)
Adolescent somatotype	2.8 (1.0)	2.9 (1.1)	2.5 (1.1)	2.7 (1.2)
BMI at age 18 (kg/m^2^)	20.8 (2.5)	21.1 (2.7)	20.9 (2.4)	21.1 (2.7)
Weight change since 18 (lbs)	26.7 (25.8)	26.3 (25.7)	29.7 (25.4)	29.7 (26.8)
Age at menarche	12.3 (1.4)	12.4 (1.4)	12.5 (1.4)	12.6 (1.4)
Parity (among parous)	2.4 (1.0)	2.5 (1.0)	3.2 (1.5)	3.2 (1.5)
Age at first birth (among parous)	26.3 (4.4)	25.9 (4.1)	25.6 (3.7)	25.2 (3.5)
Breastfeeding (months) (among parous women who ever breastfed)	16.3 (10.6)	17.1 (11.1)	12.6 (10.8)	11.4 (9.5)
Birth index	46.0 (26.4)	46.7 (25.9)	56.4 (37.4)	55.8 (37.7)
Height (inches)	65.1 (2.6)	64.9 (2.5)	64.5 (2.3)	64.6 (2.4)
Alcohol use (g/day)	4.2 (6.9)	4.2 (6.3)	5.0 (7.5)	4.8 (7.8)
Age at menopause (y)			47.8 (5.8)	46.7 (6.5)
Percent density	46.1 (18.7)	39.7 (19.0)	29.2 (17.6)	24.8 (17.2)
Dense area (cm^2^)	96.8 (57.5)	81.3 (50.8)	50.7 (38.4)	47.1 (38.9)
Non-dense area (cm^2^)	121.4 (77.0)	134.4 (80.4)	141.3 (90.3)	157.5 (91.1)
N (percent)				
Nulliparous	81 (14.5)	228 (13.2)	65 (8.9)	139 (8.2)
Ever breastfed (among parous)	361 (76.5)	1136 (76.9)	389 (53.6)	940 (55.8)
Personal history of BBD				
Confirmed by biopsy	154 (27.5)	287 (16.6)	193 (26.4)	382 (22.5)
Unconfirmed/unknown	170 (30.4)	566 (32.8)	190 (26.0)	423 (25.0)
Family history of breast cancer	67 (12.0)	146 (8.5)	132 (18.1)	225 (13.3)
Hormone therapy use^a^				
Never			191 (26.1)	547 (32.3)
Current			401 (54.9)	789 (46.5)
Past			139 (19.0)	359 (21.2)

Number of individuals missing data on the following variables: breastfeeding (*n* = 42), birth index (*n* = 169), alcohol use (*n* = 275), and age at menopause (*n* = 17, postmenopausal only)

*BMI* body mass index, *BBD* benign breast disease

^a^Matching factor (matched at blood collection)

Among premenopausal women, greater adolescent somatotype (OR per 1-unit increase = 0.90; 95 % CI: 0.82, 0.99), BMI at age 18 (OR per 5-unit increase = 0.80; 95 % CI: 0.65, 0.97), birth index (OR per 102-unit increase [which represents a comparison of a woman with four births at ages at 20, 23, 26, and 29 to a nulliparous woman] = 0.66; 95 % CI: 0.43,1.01), and age at menarche (OR per 2-year increase = 0.85; 95 % CI: 0.74, 0.98) were inversely associated with breast cancer risk (Table 2). While age at menarche was not mediated by percent MD, both adolescent somatotype and BMI at age 18 were mediated by percent MD (percent mediated = 73 % and 82 %, *p* = 0.05 and 0.04 respectively). Though not statistically significant at the 5 % level, there was some evidence that both dense area and non-dense area mediated the associations with adolescent body size (percent mediated = 22 %, *p* = 0.09 for dense area and percent mediated = 35 %, *p* = 0.11 for non-dense area) and BMI at age 18 (percent mediated = 18 %, *p* = 0.10 for dense area and percent mediated = 40 %, *p* = 0.11 for non-dense area). When we adjusted for current BMI in a sensitivity analysis, the percent mediated by percent MD for adolescent somatotype and BMI at age 18 was lower (percent mediated = 35 % and 24 %, *p* = 0.08 and 0.08 respectively). While not statistically significant, there was some evidence that the association between birth index and breast cancer risk in premenopausal women was partially mediated by percent MD (percent mediated = 38 %, *p* = 0.08). Among premenopausal women, greater age at first birth (OR per 5-year increase = 1.18; 95 % CI: 1.03, 1.36), greater height (OR per 3-inch increase = 1.14; 95 % CI: 1.02, 1.28), family history of breast cancer (OR = 1.47; 95 % CI: 1.07, 2.01), and history of biopsy-confirmed BBD (OR = 2.04; 95 % CI: 1.59, 2.62) were significantly positively associated with breast cancer risk. History of biopsy-confirmed BBD was significantly mediated by percent MD (percent mediated = 17 %, *p* = <0.01). Dense area significantly mediated the association with history of biopsy-confirmed BBD (percent mediated = 18 %, *p* < 0.01) whereas non-dense area did not mediate the association (percent mediated = 3 %, *p* = 0.07). The associations between breast cancer risk and age at first birth, height, and family history of breast cancer were not mediated by percent MD (percent mediated <5 %, *p* ≥ 0.58). Childhood somatotype, weight change since age 18, current BMI, parity, breastfeeding, alcohol use, and history of unconfirmed BBD were not significantly associated with breast cancer risk (*p* > 0.05) in this subgroup of premenopausal women.

**Table 2:** Odds ratios (ORs) and 95 % confidence intervals (95 % CIs) for breast cancer risk unadjusted and adjusted for mammographic density (MD) measures and percent of the association with breast cancer mediated by MD measures in women premenopausal at mammogram in the NHS/NHSII (*N* = 559 cases/1727 controls)

Selected risk factor	OR (95 % CI) **not** adjusted for MD	OR (95 % CI) adjusted for MD	Percent mediated by MD (*p* value)
Childhood somatotype^a^ Per 1-unit increase			
Percent MD^c^	0.93 (0.86,1.01)	0.98 (0.90,1.07)	71 % (*p* = 0.12)
Dense area^c^	0.93 (0.86,1.01)	0.95 (0.88,1.04)	30 % (*p* = 0.14)
Non-dense area^c^	0.93 (0.86,1.01)	0.96 (0.88,1.04)	35 % (*p* = 0.16)
Adolescent somatotype^a^ Per 1-unit increase			
Percent MD^c^	0.90 (0.82,0.99)	0.97 (0.88,1.07)	73 % (*p* = 0.05)
Dense area^c^	0.90 (0.82,0.99)	0.92 (0.84,1.01)	22 % (*p* = 0.09)
Non-dense area^c^	0.90 (0.82,0.99)	0.94 (0.85,1.03)	35 % (*p* = 0.11)
BMI at age 18 (kg/m^2^)^a^ Per 5-unit increase			
Percent MD^c^	0.80 (0.65,0.97)	0.96 (0.78,1.19)	82 % (*p* = 0.04)
Dense area^c^	0.80 (0.65,0.97)	0.83 (0.68,1.02)	18 % (*p* = 0.10)
Non-dense area^c^	0.80 (0.65,0.97)	0.87 (0.70,1.08)	40 % (*p* = 0.11)
Weight change since 18^b^ Per 20-lb increase			
Percent MD^c^	1.03 (0.96,1.12)	1.16 (1.06,1.26)	Not mediated^d^
Dense area^c^	1.03 (0.96,1.12)	1.05 (0.97,1.13)	Not mediated^d^
Non-dense area^c^	1.03 (0.96,1.12)	1.12 (1.02,1.23)	Not mediated^d^
BMI (kg/m^2^) Per 5-unit increase			
Percent MD^c^	1.03 (0.92,1.16)	1.22 (1.07,1.39)	Not mediated^d^
Dense area^c^	1.03 (0.92,1.16)	1.05 (0.93,1.19)	Not mediated^d^
Non-dense area^c^	1.03 (0.92,1.16)	1.16 (1.01,1.33)	Not mediated^d^
Height Per 3-inch increase			
Percent MD^c^	1.14 (1.01,1.28)	1.14 (1.01,1.29)	Not mediated^d^
Dense area^c^	1.14 (1.01,1.28)	1.13 (1.00,1.27)	6 % (*p* = 0.43)
Non-dense area^c^	1.14 (1.01,1.28)	1.15 (1.02,1.30)	Not mediated^d^
Age at menarche Per 2-year increase			
Percent MD^c^	0.85 (0.74,0.98)	0.83 (0.72,0.96)	Not mediated^d^
Dense area^c^	0.85 (0.74,0.98)	0.83 (0.72,0.96)	Not mediated^d^
Non-dense area^c^	0.85 (0.74,0.98)	0.84 (0.73,0.97)	Not mediated^d^
Nulliparous Nulliparous vs parous			
Percent MD^c^	1.15 (0.86,1.52)	1.07 (0.80,1.42)	52 % (*p* = 0.35)
Dense area^c^	1.15 (0.86,1.52)	1.11 (0.84,1.48)	23 % (*p* = 0.43)
Non-dense area^c^	1.15 (0.86,1.52)	1.11 (0.84,1.48)	22 % (*p* = 0.37)
Birth index Per 102-unit increase			
Percent MD^c^	0.66 (0.43,1.01)	0.77 (0.50,1.19)	38 % (*p* = 0.08)
Dense area^c^	0.66 (0.43,1.01)	0.69 (0.45,1.07)	13 % (*p* = 0.21)
Non-dense area^c^	0.66 (0.43,1.01)	0.71 (0.46,1.09)	18 % (*p* = 0.11)
Parity (among parous) Per one-child increase			
Percent MD^c^	1.00 (0.90,1.12)	1.03 (0.92,1.16)	Not mediated^d^
Dense area^c^	1.00 (0.90,1.12)	1.02 (0.91,1.14)	Not mediated^d^
Non-dense area^c^	1.00 (0.90,1.12)	1.01 (0.90,1.13)	Not mediated^d^
Age at first birth (among parous) Per 5-year increase			
Percent MD^c^	1.18 (1.03,1.36)	1.18 (1.02,1.36)	3 % (*p* = 0.58)
Dense area^c^	1.18 (1.03,1.36)	1.19 (1.03,1.38)	Not mediated^d^
Non-dense area^c^	1.18 (1.03,1.36)	1.18 (1.02,1.36)	3 % (*p* = 0.28)
Breastfeeding (among parous) Ever vs never			
Percent MD^c^	0.99 (0.76,1.28)	1.00 (0.77,1.30)	89 % (*p* = 0.92)
Dense area^c^	0.99 (0.76,1.28)	0.98 (0.75,1.28)	Not mediated^d^
Non-dense area^c^	0.99 (0.76,1.28)	1.00 (0.77,1.30)	75 % (*p* = 0.92)
Breastfeeding (among parous who ever breastfed) Per 12-month increase			
Percent MD^c^	0.95 (0.81,1.11)	0.93 (0.80,1.08)	Not mediated^d^
Dense area^c^	0.95 (0.81,1.11)	0.93 (0.79,1.08)	Not mediated^d^
Non-dense area^c^	0.95 (0.82,1.11)	0.95 (0.82,1.11)	1 % (*p* = 0.92)
Alcohol use Per 10 g/day increase			
Percent MD^c^	0.98 (0.84,1.15)	0.98 (0.84,1.14)	Not mediated^d^
Dense area^c^	0.98 (0.84,1.15)	0.97 (0.83,1.14)	Not mediated^d^
Non-dense area^c^	0.98 (0.84,1.15)	0.98 (0.84,1.15)	Not mediated^d^
Family history of breast cancer Yes vs no			
Percent MD^c^	1.47 (1.07,2.01)	1.46 (1.06,2.00)	2 % (*p* = 0.76)
Dense area^c^	1.47 (1.07,2.01)	1.39 (1.01,1.92)	14 % (*p* = 0.12)
Non-dense area^c^	1.47 (1.07,2.01)	1.51 (1.10,2.07)	Not mediated^d^
History of biopsy-confirmed BBD Yes vs no			
Percent MD^c^	2.04 (1.59,2.62)	1.81 (1.40,2.32)	17 % (*p* < 0.01)
Dense area^c^	2.04 (1.59,2.62)	1.80 (1.40,2.32)	18 % (*p* < 0.01)
Non-dense area^c^	2.04 (1.59,2.62)	2.00 (1.56,2.57)	3 % (*p* = 0.07)
History of unconfirmed BBD Yes vs no			
Percent MD^c^	1.12 (0.89,1.41)	1.03 (0.82,1.30)	73 % (*p* = 0.34)
Dense area^c^	1.12 (0.89,1.41)	1.01 (0.81,1.28)	87 % (*p* = 0.33)
Non-dense area^c^	1.12 (0.89,1.41)	1.11 (0.88,1.39)	9 % (*p* = 0.44)

Adjusted for age (continuous), fasting status (no, yes), time of blood collection (12 am–5:59 am, 6:00 am–7:59 am, 8:00 am–11:59 pm), mammography batch (NHS batch 1, NHS batch 2, NHSII), current BMI (continuous), BMI at age 18 (continuous), adolescent somatotype (continuous), history of biopsy-confirmed BBD (no, yes), history of unconfirmed BBD (no, yes), nulliparity (no, yes), parity (continuous), age at first birth (continuous, nulliparous set to median), and age at menarche (continuous). Percent of the total association (on the log odds scale) between the exposure and breast cancer risk that was mediated by MD was calculated using the following equation: 1-(lnOR_adjusted_/lnOR_unadjusted_)

*BMI* body mass index, *BBD* benign breast disease

^a^Not adjusted for adolescent somatotype, BMI at age 18, or current BMI

^b^Not adjusted for current BMI

^c^Square-root transformed

^d^Percent mediated calculated to be <0 %

Bold data in the header of column 2 in order to distinguish column 2 from column 3

Among postmenopausal women, greater childhood somatotype (OR per 1-unit increase = 0.89; 95 % CI: 0.83, 0.96) and greater adolescent somatotype (OR per 1-unit increase = 0.86; 95 % CI: 0.80, 0.93) were significantly inversely associated with breast cancer risk (Table 3). The associations with breast cancer risk for both of these early life body size measures were significantly mediated by percent MD (percent mediated = 26 % and *p* = 0.01 for both measures). Dense area significantly mediated the association for both childhood and adolescent somatotype (percent mediated = 16 %, *p* = 0.02 and percent mediated = 13 %, *p* = 0.01, respectively). While there was some evidence that non-dense area also mediated the association, it was not statistically significant (percent mediated = 10 %, *p* = 0.10 and percent mediated = 10 %, *p* = 0.13, respectively). After adjustment for current BMI, both of these associations were mediated by percent MD, though the percent mediated was somewhat lower (percent mediated = 19 % and 18 %, *p* = 0.01 and *p* < 0.01, respectively). Greater age at first birth, greater months breastfeeding among those who ever breastfed, family history of breast cancer, history of biopsy-confirmed BBD, greater age at menopause, and current HT use were positively associated with breast cancer risk (*p* < 0.05). The association between history of biopsy-confirmed BBD and breast cancer risk was mediated by percent MD (percent mediated = 33 %, *p* = 0.04). There was some evidence that dense area mediated the association with BBD (percent mediated = 24 %, *p* = 0.05), but less evidence for non-dense area (percent mediated = 8 %, *p* = 0.12). In addition, percent MD significantly mediated the association with breast cancer risk for current HT use (percent mediated = 22 %, *p* < 0.01). Further, both dense area and non-dense area mediated the association with current HT use (percent mediated = 14 %, *p* < 0.01 and percent mediated = 7 %, *p* = 0.02, respectively). In addition, there was some evidence that the association with greater age at first birth was partially mediated by percent MD (percent mediated = 13 %, *p* = 0.05). The associations between breast cancer risk and greater months breastfeeding among those who ever breastfed, family history of breast cancer, and greater age at menopause were not significantly mediated by percent MD (percent mediated ≤5 %, *p* ≥ 0.22). Current BMI, BMI at age 18, weight change since age 18, age at menarche, parity, birth index, breastfeeding (ever/never), height, alcohol use, history of unconfirmed BBD, and past HT use were not significantly associated with breast cancer risk in this sample of postmenopausal women. In secondary analyses, we observed little evidence of interaction between the selected risk factors and percent MD on breast cancer risk. Significant interaction (*p* ≤ 0.05) was only observed for birth index, breastfeeding among women who ever breastfed, and height (Additional file 3: Table S3 and Additional file 4: Table S4). Modeling interaction between the exposures and percent MD produced similar results to our primary analysis, though the estimates of percent mediated in premenopausal women did vary by the level of percent MD in the referent category for birth index (Additional file 3: Table S3). In this secondary analysis, a substantial portion of the effect of adolescent somatotype and of BMI at age 18 among premenopausal women was mediated by percent MD and suggests that this mediation was robust to potential unmeasured confounding by common causes of percent MD and breast cancer. To completely account for the mediated effect for adolescent somatotype among premenopausal women, an unmeasured confounder that increased percent MD by two-thirds of a standard deviation would have to increase breast cancer risk by at least 2.1-fold. To completely account for the mediated effect for BMI at age 18 among premenopausal women, an unmeasured confounder that increased percent MD by two-thirds of a standard deviation would have to increase risk by at least 2.8-fold. Substantial unmeasured confounding would thus be required to completely explain away these mediated effects of adolescent somatotype and of BMI at age 18 among premenopausal women.

**Table 3:** Odds ratios (ORs) and 95 % confidence intervals (95 % CIs) for breast cancer risk unadjusted and adjusted for mammographic density (MD) measures and percent of the association with breast cancer mediated by MD measures in women postmenopausal at mammogram in the NHS/NHSII (*N* = 731 cases/1695 controls)

Selected risk factor	OR (95 % CI) **not** adjusted for MD	OR (95 % CI) adjusted for MD	Percent mediated by MD (*p* value)
Childhood somatotype^a^ Per 1-unit increase			
Percent MD^c^	0.89 (0.83,0.96)	0.92 (0.85,0.99)	26 % (*p* = 0.01)
Dense area^c^	0.89 (0.83,0.96)	0.91 (0.84,0.98)	16 % (*p* = 0.02)
Non-dense area^c^	0.89 (0.83,0.96)	0.90 (0.84,0.97)	10 % (*p* = 0.10)
Adolescent somatotype^a^ Per 1-unit increase			
Percent MD^c^	0.86 (0.80,0.93)	0.90 (0.83,0.97)	26 % (*p* = 0.01)
Dense area^c^	0.86 (0.80,0.93)	0.88 (0.81,0.95)	13 % (*p* = 0.01)
Non-dense area^c^	0.86 (0.80,0.93)	0.88 (0.81,0.95)	10 % (*p* = 0.13)
BMI at age 18 (kg/m^2^)^a^ Per 5-unit increase			
Percent MD^c^	0.88 (0.74,1.05)	1.00 (0.83,1.19)	98 % (*p* = 0.17)
Dense area^c^	0.88 (0.74,1.05)	0.92 (0.77,1.10)	35 % (*p* = 0.19)
Non-dense area^c^	0.88 (0.74,1.05)	0.94 (0.78,1.13)	51 % (*p* = 0.24)
Weight change since 18^b^ Per 20-lb increase			
Percent MD^c^	1.03 (0.96,1.10)	1.11 (1.03,1.19)	Not mediated^d^
Dense area^c^	1.03 (0.96,1.10)	1.04 (0.97,1.12)	Not mediated^d^
Non-dense area^c^	1.03 (0.96,1.10)	1.10 (1.01,1.19)	Not mediated^d^
BMI (kg/m^2^) Per 5-unit increase			
Percent MD^c^	1.05 (0.95,1.16)	1.17 (1.05,1.30)	Not mediated^d^
Dense area^c^	1.05 (0.95,1.16)	1.07 (0.96,1.18)	Not mediated^d^
Non-dense area^c^	1.05 (0.95,1.16)	1.16 (1.02,1.30)	Not mediated^d^
Height Per 3-inch increase			
Percent MD^c^	0.95 (0.85,1.06)	0.96 (0.86,1.07)	22 % (*p* = 0.41)
Dense area^c^	0.95 (0.85,1.06)	0.95 (0.85,1.06)	Not mediated^d^
Non-dense area^c^	0.95 (0.85,1.06)	0.96 (0.86,1.07)	20 % (*p* = 0.39)
Age at menarche Per 2-year increase			
Percent MD^c^	0.92 (0.80,1.05)	0.93 (0.81,1.06)	11 % (*p* = 0.37)
Dense area^c^	0.92 (0.80,1.05)	0.93 (0.81,1.06)	13 % (*p* = 0.31)
Non-dense area^c^	0.92 (0.80,1.05)	0.92 (0.80,1.05)	Not mediated^d^
Nulliparous Nulliparous vs parous			
Percent MD^c^	1.22 (0.88,1.69)	1.12 (0.80,1.56)	43 % (*p* = 0.26)
Dense area^c^	1.22 (0.88,1.69)	1.14 (0.82,1.59)	32 % (*p* = 0.28)
Non-dense area^c^	1.22 (0.88,1.69)	1.19 (0.86,1.65)	11 % (*p* = 0.36)
Birth index Per 102-unit increase			
Percent MD^c^	0.96 (0.73,1.25)	1.04 (0.79,1.36)	Not mediated^e^
Dense area^c^	0.96 (0.73,1.25)	1.01 (0.78,1.33)	Not mediated^e^
Non-dense area^c^	0.96 (0.73,1.25)	0.97 (0.74,1.27)	38 % (*p* = 0.75)
Parity (among parous) Per one-child increase			
Percent MD^c^	1.02 (0.95,1.09)	1.03 (0.96,1.11)	Not mediated^d^
Dense area^c^	1.02 (0.95,1.09)	1.03 (0.96,1.10)	Not mediated^d^
Non-dense area^c^	1.02 (0.95,1.09)	1.02 (0.95,1.09)	Not mediated^d^
Age at first birth (among parous) Per 5-year increase			
Percent MD^c^	1.23 (1.07,1.41)	1.19 (1.04,1.38)	13 % (*p* = 0.05)
Dense area^c^	1.23 (1.07,1.41)	1.20 (1.05,1.39)	9 % (*p* = 0.09)
Non-dense area^c^	1.23 (1.07,1.41)	1.21 (1.05,1.40)	5 % (*p* = 0.15)
Breastfeeding (among parous) Ever vs never			
Percent MD^c^	0.96 (0.79,1.16)	0.97 (0.80,1.17)	22 % (*p* = 0.70)
Dense area^c^	0.96 (0.79,1.16)	0.96 (0.79,1.17)	11 % (*p* = 0.74)
Non-dense area^c^	0.96 (0.79,1.16)	0.97 (0.80,1.17)	19 % (*p* = 0.68)
Breastfeeding (among parous women who ever breastfed) Per 12-month increase			
Percent MD^c^	1.25 (1.06,1.46)	1.25 (1.06,1.47)	Not mediated^d^
Dense area^c^	1.25 (1.06,1.46)	1.24 (1.06,1.46)	2 % (*p* = 0.57)
Non-dense area^c^	1.25 (1.06,1.46)	1.26 (1.07,1.48)	Not mediated^d^
Alcohol use Per 10 g/day increase			
Percent MD^c^	1.01 (0.90,1.14)	1.00 (0.89,1.13)	73 % (*p* = 0.85)
Dense area^c^	1.01 (0.90,1.14)	1.01 (0.90,1.13)	41 % (*p* = 0.85)
Non-dense area^c^	1.01 (0.90,1.14)	1.01 (0.90,1.13)	38 % (*p* = 0.85)
Family history of breast cancer Yes vs no			
Percent MD^c^	1.45 (1.15,1.85)	1.45 (1.14,1.84)	1 % (*p* = 0.78)
Dense area^c^	1.45 (1.15,1.85)	1.44 (1.14,1.84)	2 % (*p* = 0.52)
Non-dense area^c^	1.45 (1.15,1.85)	1.46 (1.15,1.86)	Not mediated^d^
History of biopsy-confirmed BBD Yes vs no			
Percent MD^c^	1.29 (1.04,1.61)	1.19 (0.95,1.48)	33 % (*p* = 0.04)
Dense area^c^	1.29 (1.04,1.61)	1.22 (0.98,1.52)	24 % (*p* = 0.05)
Non-dense area^c^	1.29 (1.04,1.61)	1.27 (1.02,1.58)	8 % (*p* = 0.12)
History of unconfirmed BBD Yes vs no			
Percent MD^c^	1.19 (0.95,1.48)	1.13 (0.90,1.41)	29 % (*p* = 0.17)
Dense area^c^	1.19 (0.95,1.48)	1.13 (0.91,1.41)	28 % (*p* = 0.17)
Non-dense area^c^	1.19 (0.95,1.48)	1.19 (0.95,1.48)	0 % (*p* = 0.97)
Age at menopause Per 4-year increase			
Percent MD^c^	1.12 (1.05,1.20)	1.12 (1.04,1.19)	5 % (*p* = 0.22)
Dense area^c^	1.12 (1.05,1.20)	1.12 (1.05,1.20)	3 % (*p* = 0.30)
Non-dense area^c^	1.12 (1.05,1.20)	1.12 (1.05,1.20)	3 % (*p* = 0.20)
Hormone therapy use Past vs never			
Percent MD^c^	1.18 (0.91,1.53)	1.12 (0.86,1.46)	31 % (*p* = 0.26)
Dense area^c^	1.18 (0.91,1.53)	1.15 (0.88,1.50)	14 % (*p* = 0.31)
Non-dense area^c^	1.18 (0.91,1.53)	1.15 (0.88,1.50)	13 % (*p* = 0.31)
Hormone therapy use Current vs never			
Percent MD^c^	1.71 (1.37,2.13)	1.52 (1.22,1.90)	22 % (*p* < 0.01)
Dense area^c^	1.71 (1.37,2.13)	1.58 (1.27,1.98)	14 % (*p* < 0.01)
Non-dense area^c^	1.71 (1.37,2.13)	1.65 (1.32,2.05)	7 % (*p* = 0.02)

Adjusted for age (continuous), fasting status (no, yes), time of blood collection (12 am–5:59 am, 6:00 am–7:59 am, 8:00 am–11:59 pm), mammography batch (NHS batch 1, NHS batch 2, NHSII), current BMI (continuous), BMI at age 18 (continuous), adolescent somatotype (continuous), history of biopsy-confirmed BBD (no, yes), history of unconfirmed BBD (no, yes), nulliparity (no, yes), parity (continuous), age at first birth (continuous, nulliparous set to median), age at menarche (continuous), and HT use (never, past, current). Percent of the total association (on the log odds scale) between the exposure and breast cancer risk that was mediated by MD was calculated using the following equation: 1-(lnOR_adjusted_/lnOR_unadjusted_)

*BMI* body mass index, *BBD* benign breast disease

^a^Not adjusted for adolescent somatotype, BMI at age 18, or current BMI

^b^Not adjusted for current BMI

^c^Square-root transformed

^d^Percent mediated calculated to be <0 %

^e^Percent mediated calculated to be >100 %

Bold data in the header of column 2 in order to distinguish column 2 from column 3

## Discussion

Among premenopausal women, we observed that percent MD significantly mediated the associations of adolescent somatotype and BMI at age 18 with breast cancer risk. In postmenopausal women, associations of early life body size measures with breast cancer risk, specifically childhood and adolescent somatotype, were also significantly mediated by percent MD, though the percent mediated was more modest than in premenopausal women. Further, the association between current HT use and breast cancer risk was also significantly mediated by percent MD in postmenopausal women. The associations between personal history of biopsy-confirmed BBD and breast cancer risk were significantly mediated by percent MD in both pre- and postmenopausal women. The associations of other risk factors, such as age at menarche, age at first birth, height, family history of breast cancer, and age at menopause with breast cancer risk were not mediated by percent MD.

Several prior studies have observed that greater body fatness in childhood and adolescence is associated with lower risk of breast cancer in adulthood [23–28] as well as lower percent MD in adulthood [6, 10, 29–34]. Some studies have examined whether the association between early life body size and breast cancer risk is mediated by MD, however none have attempted to quantify the extent to which the associations are mediated by MD. In a prior study in the NHS/NHSII, the associations between childhood somatotype, adolescent somatotype, and BMI at age 18 and breast cancer risk were partially attenuated when models were adjusted for percent MD, consistent with our current study [10]. In addition, a study of over 13,000 women 50 years of age or older in Denmark observed that childhood BMI was inversely associated with risk of breast cancer [33]. When adjusted for a binary classification of MD (fatty breasts vs mixed/dense breasts), these associations were attenuated. For example, the hazard ratio (HR) for the association between the BMI at age 7 (per z-score) and breast cancer risk was 0.91 (95 % CI: 0.83–0.99) in age and birth cohort adjusted models, whereas the HR was 0.97 (95 % CI: 0.88–1.06) after further adjusting for MD. However, this study had several limitations including a binary classification of MD as well as limited ability to adjust for potential confounders. While prior studies have observed that history of BBD and percent MD are independent risk factors for breast cancer [35], to our knowledge no studies have examined the extent to which the association between BBD and breast cancer is mediated by MD. In the current study, we observed that the association between biopsy-confirmed BBD and breast cancer risk was attenuated after adjustment for percent MD. However, while we used information on BBD diagnosis prior to the mammogram date, it is likely that women with BBD had higher percent MD at the time of the biopsy compared to women without BBD. Further, some studies suggest that high percent MD may lead to the development of incident BBD [36]. Therefore, percent MD is unlikely be a downstream consequence of BBD, but rather a co-morbidity of, or a risk factor for, BBD [35, 36]. Previous research has observed that HT use is associated with greater percent MD [37, 38] as well as an increased risk of breast cancer [39]. In an analysis of over 1700 postmenopausal women from three case-control studies, current hormone therapy use was associated with an increased risk of breast cancer (OR = 1.26, 95 % CI: 1.00–1.59), which was partially attenuated after adjustment for percent MD (OR = 1.19, 95 % CI: 0.94–1.51) [11]. Interestingly, this attenuation of 25 % is very similar to our observation that the percent mediated for association with current HT use was 22 %.

Our study has several limitations. The associations between exposures and risk, as well as the proportion of the associations that are mediated by MD, may differ by breast cancer subtype (e.g., ER status). However, we had insufficient power in this analysis to examine the proportion mediated by MD according to breast cancer subtype. Further, we were unable to further stratify by menopausal status at diagnosis due to limited power and were able to measure MD at only one point in time. All exposure data is self-reported, which may result in some misclassification. However, data on all exposures was collected prior to both the mammogram and breast cancer diagnosis, therefore any misclassification should be non-differential with respect to both MD measurements and case-control status. While MD measures are highly reproducible, there is the possibility for random error. As the mammogram reader was blinded to both exposure and case-control status, any misclassification of MD should be non-differential. Non-differential measurement error in the mediator tends to bias the mediated effects toward the null so the true proportion mediated measures may in fact be larger than those reported here [22, 40]. Due to the limitations of our data, the effect estimates between risk factors and breast cancer risk should be interpreted as associations and our estimates of mediation by percent MD should be interpreted as statistical mediation and not necessarily causal. However, the results for the mediated effect for adolescent somatotype and for BMI at age 18 among premenopausal women seemed robust to potential unmeasured confounding of MD and breast cancer risk. The NHS/NHSII may have different distributions of risk factors compared to other US populations (e.g., women in the NHS were married at enrollment and were more likely to be parous). Therefore, confirmation of these results in other cohorts is warranted. The strengths of our study include the centralized collection and reading of mammograms, the first quantitative assessment of mediation by MD on breast cancer risk, and detailed adjustment for potential confounders of the relationships between the exposure and mediator, the exposure and outcome, and the mediator and outcome.

## Conclusions

Overall, we observed that percent MD partially mediated some of the associations between established breast cancer risk factors and breast cancer risk, though the magnitude varied by risk factor and menopausal status. In particular, early life body size was significantly mediated in both pre- and postmenopausal women as was HT use in postmenopausal women. This work suggests that the utility of percent MD as an intermediate marker of breast cancer risk may depend on the exposure under study. Additional research is necessary to confirm these observations as well as to explore whether the extent to which MD mediates the associations with established breast cancer risk factors varies by breast cancer subtype.

## Additional files

Additional file 1: Table S1. Selected risk factors (at time of mammography) by quartiles of percent mammographic density and menopausal status at mammogram among controls (NHS/NHSII). (DOC 61 kb) Additional file 2: Table S2. Differences in mammographic density measures by breast cancer risk factors in 1727 premenopausal and 1695 postmenopausal controls (NHS/NHSII). (DOC 65 kb) Additional file 3: Table S3. Mediation analysis in women **premenopausal** at mammogram, modeling interaction between the exposure and percent MD (NHS/NHS2). (DOC 108 kb) Additional file 4: Table S4. Mediation analysis in women **postmenopausal** at mammogram, modeling interaction between the exposure and percent MD (NHS/NHS2). (DOC 124 kb)

## Abbreviations

BBD: Benign breast disease
BMI: Body mass index
HT: Hormone therapy
MD: Mammographic density
NHS: Nurses’ Health Study
