# Supplementary material for: Mammographic density and breast cancer risk: a mediation analysis
Source: Breast Cancer Res. 2016 Sep 21;18:94. doi: 10.1186/s13058-016-0750-0 (PMC5031307; doi:10.1186/s13058-016-0750-0)
Supplement: Additional file 1: Table S1. — Selected risk factors (at time of mammography) by quartiles of percent mammographic density and menopausal status at mammogram among controls (NHS/NHSII). (DOC 61 kb) [file 13058_2016_750_MOESM1_ESM.doc]

**Additional file 1: Table S1. Selected risk factors (at time of mammography) by quartiles of percent mammographic density and menopausal status at mammogram among controls (NHS/NHSII)**

|  | **Premenopausal** | | | | **Postmenopausal** | | | |
| --- | --- | --- | --- | --- | --- | --- | --- | --- |
|  | **Quartile 1 (<25)**  **N=431** | **Quartile 2 (25-<39) N=432** | **Quartile 3 (39-<54) N=432** | **Quartile 4 (54+)**  **N=432** | **Quartile 1 (<11)**  **N=423** | **Quartile 2 (11-<22) N=425** | **Quartile 3 (22-<36) N=422** | **Quartile 4 (36+)**  **N=425** |
| **Mean (SD)** |  | | | |  | | | |
| Age* | 47.0(4.4) | 46.2(4.3) | 45.9(4.3) | 45.3(4.2) | 61.6(6.3) | 60.4(7.3) | 59.3(7.7) | 57.2(7.7) |
| Current BMI (kg/m^2^) | 29.3(5.5) | 25.7(4.7) | 24.2(4.0) | 22.5(3.2) | 29.3(5.4) | 26.5(4.7) | 25.1(4.4) | 23.6(3.8) |
| Childhood somatotype | 3.0(1.3) | 2.6(1.1) | 2.4(1.2) | 2.3(1.1) | 2.9(1.4) | 2.3(1.4) | 2.2(1.2) | 2.2(1.1) |
| Adolescent somatotype | 3.4(1.1) | 2.9(1.0) | 2.7(1.0) | 2.6(0.9) | 3.2(1.3) | 2.6(1.2) | 2.5(1.1) | 2.5(1.0) |
| BMI at age 18 (kg/m^2^) | 22.5(3.0) | 21.1(2.5) | 20.6(2.4) | 20.1(1.9) | 22.4(3.1) | 21.1(2.6) | 20.6(2.4) | 20.3(2.0) |
| Weight change since 18 (lbs) | 40.7(30.1) | 28.0(23.7) | 22.0(22.0) | 14.5(18) | 40.8(29.4) | 31.5(24.9) | 26.3(25.6) | 20.1(22.6) |
| Age at menarche | 12.1(1.3) | 12.3(1.3) | 12.5(1.4) | 12.7(1.5) | 12.6(1.4) | 12.4(1.3) | 12.6(1.5) | 12.6(1.3) |
| Parity (among parous) | 2.6(1.1) | 2.5(1.0) | 2.5(1.0) | 2.3(0.8) | 3.5(1.5) | 3.3(1.6) | 3.2(1.5) | 2.8(1.4) |
| Age at first birth (among parous) | 25.1(4.0) | 26.2(3.9) | 26.2(4.1) | 26.3(4.1) | 25.1(3.1) | 24.9(3.3) | 25.1(3.6) | 25.8(3.9) |
| Breastfeeding (months) | 16.4(11.2) | 17.5(11.1) | 18.5(11.4) | 15.9(10.6) | 10.5(9.2) | 11.9(9.9) | 12.1(9.9) | 11.2(9.1) |
| (among parous women who ever breastfed) |
| Birth index | 53(29.6) | 46.6(24.2) | 46.4(26.4) | 40.4(20.6) | 62.3(38.1) | 61(37.6) | 54.3(37.6) | 45.6(35.4) |
| Height (inches) | 64.8(2.5) | 64.7(2.7) | 65.0(2.4) | 65(2.4) | 64.6(2.4) | 64.4(2.3) | 64.7(2.4) | 64.7(2.4) |
| Alcohol use (g/day) | 3.4(5.3) | 4(6.5) | 4.6(6.7) | 4.7(6.6) | 5.1(8.7) | 3.9(6.3) | 4.5(7.6) | 5.8(8.2) |
| Age at menopause |  |  |  |  | 47.4(6.3) | 47.1(6.4) | 46.3(6.6) | 46(6.6) |
| **N (Percent)** |  |  |  |  |  |  |  |  |
| Nulliparous | 43(10.0) | 52(12.0) | 57(13.2) | 76(17.6) | 26(6.1) | 22(5.2) | 37(8.8) | 54(12.7) |
| Ever breastfed (among parous) | 279(65) | 300(70.8) | 287(67.7) | 270(62.9) | 223(53.3) | 258(60.7) | 236(56.5) | 223(52.6) |
| History of BBD |  |  |  |  |  |  |  |  |
| Biopsy-confirmed | 51(11.8) | 61(14.1) | 75(17.4) | 100(23.1) | 68(16.1) | 92(21.6) | 112(26.5) | 110(25.9) |
| Unconfirmed/unknown | 126(29.2) | 132(30.6) | 146(33.8) | 162(37.5) | 80(18.9) | 105(24.7) | 103(24.4) | 135(31.8) |
| Family history of breast cancer | 28(6.5) | 42(9.7) | 41(9.5) | 35(8.1) | 50(11.8) | 56(13.2) | 63(14.9) | 56(13.2) |
| Hormone therapy use* |  |  |  |  |  |  |  |  |
| Never |  |  |  |  | 195(46.1) | 157(36.9) | 110(26.1) | 85(20.0) |
| Current |  |  |  |  | 127(30.0) | 175(41.2) | 211(50.0) | 276(64.9) |
| Past |  |  |  |  | 101(23.9) | 93(21.9) | 101(23.9) | 64(15.1) |

*Matching factor (matched at blood collection)

BMI=body mass index, BBD=benign breast disease
