# Supplementary material for: Mammographic density and breast cancer risk: a mediation analysis
Source: Breast Cancer Res. 2016 Sep 21;18:94. doi: 10.1186/s13058-016-0750-0 (PMC5031307; doi:10.1186/s13058-016-0750-0)
Supplement: Additional file 2: Table S2. — Differences in mammographic density measures by breast cancer risk factors in 1727 premenopausal and 1695 postmenopausal controls (NHS/NHSII). (DOC 65 kb) [file 13058_2016_750_MOESM2_ESM.doc]

**Additional file 2: Table S2. Differences in mammographic density measures by breast cancer risk factors in 1727 premenopausal and 1695 postmenopausal controls (NHS/NHSII)**

|  | **Percent MD**^±^ | | **Dense Area^±^** | | **Non-dense area^±^** | |
| --- | --- | --- | --- | --- | --- | --- |
|  | **Premenopausal** | **Postmenopausal** | **Premenopausal** | **Postmenopausal** | **Premenopausal** | **Postmenopausal** |
| BMI (kg/m^2^)  Per 5 unit increase | **-0.68**  **(-0.75,-0.60)** | **-0.66**  **(-0.74,-0.58)** | **-0.22**  **(-0.36,-0.09)** | **-0.27**  **(-0.39,-0.15)** | **1.78**  **(1.65,1.92)** | **1.70**  **(1.57,1.83)** |
| Childhood somatotype*  Per 1 unit increase | **-0.30**  **(-0.36,-0.23)** | **-0.26**  **(-0.32,-0.20)** | **-0.24**  **(-0.34,-0.15)** | **-0.29**  **(-0.37,-0.20)** | **0.56**  **(0.45,0.68)** | **0.34**  **(0.23,0.45)** |
| Adolescent somatotype*  Per 1 unit increase | **-0.44**  **(-0.51,-0.37)** | **-0.34**  **(-0.41,-0.27)** | **-0.29**  **(-0.40,-0.18)** | **-0.31**  **(-0.40,-0.21)** | **0.90**  **(0.77,1.03)** | **0.57**  **(0.45,0.69)** |
| BMI at age 18 (kg/m^2^)*  Per 5 unit increase | **-1.01**  **(-1.15,-0.88)** | **-0.93**  **(-1.08,-0.78)** | **-0.50**  **(-0.72,-0.28)** | **-0.65**  **(-0.85,-0.44)** | **2.32**  **(2.07,2.57)** | **1.97**  **(1.71,2.22)** |
| Weight change since 18^#^  Per 20 lb increase | **-0.45**  **(-0.50,-0.40)** | **-0.44**  **(-0.49,-0.38)** | **-0.15**  **(-0.24,-0.06)** | **-0.18**  **(-0.26,-0.10)** | **1.19**  **(1.10,1.27)** | **1.14**  **(1.06,1.23)** |
| Age at menarche  Per 2 year increase | **0.10**  **(0.00,0.19)** | -0.05  (-0.16,0.06) | 0.10  (-0.07,0.27) | -0.09  (-0.25,0.06) | -0.15  (-0.31,0.01) | 0.00  (-0.17,0.17) |
| Nulliparous  Nulliparous vs parous | **0.21**  **(0.01,0.40)** | **0.53**  **(0.26,0.80)** | 0.23  (-0.11,0.57) | **0.72**  **(0.32,1.11)** | -0.18  (-0.52,0.15) | -0.33  (-0.75,0.09) |
| Birth Index  Per 102 unit increase | **-0.61**  **(-0.90,-0.33)** | **-0.41**  **(-0.63,-0.19)** | -0.45  (-0.95,0.05) | **-0.53**  **(-0.84,-0.21)** | **0.92**  **(0.44,1.41)** | 0.13  (-0.21,0.47) |
| Parity (among parous)  Per 1 child increase | **-0.11**  **(-0.19,-0.04)** | -0.04  (-0.10,0.02) | -0.09  (-0.22,0.04) | -0.05  (-0.13,0.03) | **0.22**  **(0.09,0.34)** | -0.01  (-0.10,0.08) |
| Age at first birth (among parous)  Per 5 year increase | 0.14  (-0.04,0.33) | **0.48**  **(0.25,0.71)** | 0.11  (-0.22,0.43) | **0.54**  **(0.22,0.87)** | -0.19  (-0.50,0.12) | **-0.58**  **(-0.94,-0.22)** |
| Breastfeeding (among parous)  Ever vs never | -0.05  (-0.23,0.13) | -0.03  (-0.18,0.13) | 0.10  (-0.21,0.43) | -0.03  (-0.25,0.20) | 0.20  (-0.10,0.5) | 0.12  (-0.12,0.37) |
| Breastfeeding (among parous women who ever breastfed)  Per 12 months increase | 0.04  (-0.06,0.14) | 0.06  (-0.07,0.18) | 0.10  (-0.08,0.28) | 0.10  (-0.09,0.29) | 0.04  (-0.13,0.21) | 0.07  (-0.14,0.27) |
| Height  Per 3 inch increase | 0.03  (-0.05,0.11) | -0.06  (-0.16,0.03) | 0.09  (-0.05,0.23) | 0.06  (-0.08,0.19) | 0.08  (-0.05,0.22) | **0.26**  **(0.11,0.40)** |
| Alcohol use  Per 10 g/day increase | 0.05  (-0.05,0.16) | 0.01  (-0.09,0.11) | 0.09  (-0.10,0.28) | -0.02  (-0.16,0.12) | -0.04  (-0.23,0.14) | -0.07  (-0.23,0.08) |
| Family history of breast cancer  Yes vs no | 0.05  (-0.18,0.29) | 0.05  (-0.16,0.27) | **0.43**  **(0.02,0.83)** | 0.14  (-0.18,0.45) | **0.45**  **(0.05,0.84)** | -0.01  (-0.35,0.33) |
| History of confirmed BBD  Yes vs no | **0.29**  **(0.11,0.47)** | **0.36**  **(0.18,0.53)** | **0.60**  **(0.29, 0.91)** | **0.50**  **(0.24,0.75)** | -0.15  (-0.44,0.15) | **-0.29**  **(-0.57,-0.02)** |
| History of unconfirmed BBD  Yes vs no | **0.18**  **(0.04,0.32)** | **0.16**  **(-0.01,0.33)** | **0.33**  **(0.08,0.57)** | **0.29**  **(0.04,0.53)** | -0.11  (-0.34,0.13) | -0.03  (-0.29,0.24) |
| Age at menopause  Per 4 year increase |  | 0.03  (-0.02,0.08) |  | 0.03  (-0.04,0.11) |  | -0.06  (-0.14,0.02) |
| Hormone therapy use  Past vs Never |  | **0.27**  **(0.06,0.47)** |  | 0.20  (-0.10,0.50) |  | **-0.42**  **(-0.75,-0.10)** |
| Hormone therapy use  Current vs Never |  | **0.57**  **(0.39,0.75)** |  | **0.71**  **(0.46,0.97)** |  | **-0.48**  **(-0.76,-0.21)** |

Adjusted for age (continuous), fasting status (no, yes), time of blood collection (12 am–5:59 am, 6:00 am–7:59 am, 8:00 am–11:59 pm), mammography batch (NHS batch 1, NHS batch 2, NHSII), current BMI, BMI at age 18, adolescent somatotype, history of biopsy-confirmed BBD (no, yes), history of unconfirmed BBD (no, yes), parity, age at first birth, age at menarche, and HT use (postmenopausal only: never, past, current).

*Not adjusted for adolescent somatotype, BMI at age 18, or current BMI

^#^ Not adjusted for current BMI

^Among parous

^^Among parous women who ever breastfed

±Square-root transformed
