# Supplementary material for: Mammographic density and breast cancer risk: a mediation analysis
Source: Breast Cancer Res. 2016 Sep 21;18:94. doi: 10.1186/s13058-016-0750-0 (PMC5031307; doi:10.1186/s13058-016-0750-0)
Supplement: Additional file 3: Table S3. — Mediation analysis in women premenopausal at mammogram, modeling interaction between the exposure and percent MD (NHS/NHS2). (DOC 108 kb) [file 13058_2016_750_MOESM3_ESM.doc]

**Additional file 3: Table S3.** Mediation analysis in women **premenopausal** at mammogram, modeling interaction between the exposure and percent MD (NHS/NHS2)

|  | **p-interaction**^†^ | **OR_NDE_ (95% CI)** | **OR_NIE_ (95% CI)** | **OR_TOTAL_ (95% CI)** | **Percent mediated by percent MD** |
| --- | --- | --- | --- | --- | --- |
| BMI (kg/m^2^) | 0.26 |  |  |  |  |
| 30 vs 25 |  | 1.26(1.09,1.45) | 0.83(0.77,0.88) | 1.04(0.92,1.18) | Not mediated^ |
| 25 vs 30 |  | 0.82(0.71,0.93) | 1.18(1.12,1.25) | 0.96(0.85,1.09) | Not mediated^ |
| Childhood somatotype* | 0.33 |  |  |  |  |
| 4 vs 3 |  | 0.98(0.90,1.08) | 0.93(0.90,0.96) | 0.92(0.84,1.00) | 82% |
| 3 vs 4 |  | 1.02(0.94,1.12) | 1.07(1.04,1.09) | 1.09(1.00,1.19) | 73% |
| Adolescent somatotype* | 0.18 |  |  |  |  |
| 4 vs 3 |  | 0.98(0.89,1.09) | 0.90(0.86,0.94) | 0.88(0.80,0.98) | 87% |
| 3 vs 4 |  | 1.04(0.93,1.15) | 1.10(1.06,1.13) | 1.13(1.02,1.26) | 72% |
| BMI at age 18 (kg/m^2^)* | 0.20 |  |  |  |  |
| 30 vs 25 |  | 0.95(0.76,1.19) | 0.7(0.56,0.89) | 0.67(0.5,0.91) | 88% |
| 25 vs 30 |  | 1.14(0.88,1.46) | 1.31(1.16,1.48) | 1.49(1.1,2.01) | 68% |
| Weight change since 18 (lbs)^#^ | 0.72 |  |  |  |  |
| 20 vs 0 |  | 1.17(1.05,1.30) | 0.89(0.86,0.93) | 1.04(0.94,1.16) | Not mediated^ |
| 0 vs 20 |  | 0.86(0.78,0.94) | 1.12(1.06,1.17) | 0.96(0.86,1.06) | Not mediated^ |
| Age at menarche | 0.32 |  |  |  |  |
| 14 vs 12 |  | 0.83(0.72,0.96) | 1.02(1.00,1.04) | 0.85(0.73,0.99) | Not mediated^ |
| 12 vs 14 |  | 1.21(1.04,1.40) | 0.98(0.95,1.00) | 1.18(1.02,1.37) | Not mediated^ |
| Nulliparous | 0.10 |  |  |  |  |
| Nulliparous vs parous |  | 1.03(0.74,1.43) | 1.09(0.99,1.19) | 1.12(0.80,1.57) | 75% |
| Parous vs nulliparous |  | 0.94(0.67,1.30) | 0.95(0.91,1.00) | 0.89(0.64,1.25) | 43% |
| Parity (among parous) | 0.38 |  |  |  |  |
| 2 vs 1 |  | 1.02(0.90,1.15) | 0.97(0.95,0.99) | 0.99(0.87,1.12) | Not mediated^^ |
| 1 vs 2 |  | 0.98(0.87,1.11) | 1.03(1.01,1.06) | 1.01(0.89,1.15) | Not mediated^^ |
| Age at first birth  (among parous) | 0.23 |  |  |  |  |
| 30 vs 25 |  | 1.18(1.02,1.36) | 1.01(1.00,1.03) | 1.20(1.04,1.38) | 8% |
| 25 vs 30 |  | 0.85(0.74,0.98) | 0.98(0.96,1.00) | 0.84(0.73,0.96) | 10% |
| Birth Index | 0.04 |  |  |  |  |
| 102 vs 0 |  | 0.68(0.42,1.10) | 0.92(0.84,1.01) | 0.63(0.39,1.00) | 18% |
| 0 vs 102 |  | 1.29(0.82,2.05) | 1.24(1.09,1.40) | 1.60(1.00,2.55) | 45% |
| Breastfeeding(among parous) | 0.61 |  |  |  |  |
| Ever vs never |  | 0.99(0.76,1.30) | 0.99(0.95,1.03) | 0.98(0.75,1.29) | 58% |
| Never vs ever |  | 1.01(0.77,1.31) | 1.01(0.97,1.06) | 1.02(0.78,1.34) | 69% |
| Breastfeeding (months)  (among parous women who ever breastfed) | <0.01 |  |  |  |  |
| 12 vs 1 |  | 0.92(0.79,1.08) | 1.01(0.99,1.02) | 0.93(0.80,1.08) | Not mediated^ |
| 1 vs 12 |  | 1.08(0.93,1.26) | 1.00(0.99,1.01) | 1.08(0.92,1.25) | Not mediated^ |
| Height (inches) | 0.02 |  |  |  |  |
| 63 vs 66 |  | 1.13(0.99,1.30) | 1.01(0.99,1.03) | 1.14(1.00,1.31) | 5% |
| 66 vs 63 |  | 0.88(0.77,1.01) | 0.99(0.97,1.02) | 0.88(0.76,1.00) | 7% |
| Alcohol use (g/day) | 0.72 |  |  |  |  |
| 10 vs 0 |  | 0.97(0.83,1.14) | 1.01(0.99,1.04) | 0.99(0.84,1.16) | Not mediated^ |
| 0 vs 10 |  | 1.03(0.88,1.21) | 0.99(0.96,1.01) | 1.01(0.86,1.19) | Not mediated^ |
| Family history of breast cancer | 0.57 |  |  |  |  |
| Yes vs no |  | 1.46(1.06,2.02) | 1.02(0.94,1.10) | 1.49(1.07,2.07) | 4% |
| No vs yes |  | 0.68(0.49,0.94) | 0.99(0.93,1.05) | 0.67(0.48,0.94) | 3% |
| History of confirmed BBD | 0.79 |  |  |  |  |
| Yes vs no |  | 1.80(1.39,2.33) | 1.12(1.03,1.21) | 2.01(1.55,2.60) | 16% |
| No vs yes |  | 0.55(0.43,0.71) | 0.90(0.85,0.96) | 0.50(0.38,0.65) | 14% |
| History of unconfirmed BBD | 0.95 |  |  |  |  |
| Yes vs no |  | 1.03(0.82,1.30) | 1.08(1.02,1.13) | 1.11(0.88,1.40) | 71% |
| No vs yes |  | 0.97(0.77,1.22) | 0.93(0.89,0.97) | 0.90(0.71,1.14) | 69% |

Adjusted for age (continuous), fasting status (no, yes), time of blood collection (12 am–5:59 am, 6:00 am–7:59 am, 8:00 am–11:59 pm), mammography batch (NHS batch 1, NHS batch 2, NHSII), current BMI (continuous), BMI at age 18 (continuous), adolescent somatotype (continuous), history of BBD (no, yes), nulliparity (no, yes), parity (continuous), age at first birth (continuous, nulliparous set to median), and age at menarche (continuous).

^†^ p for interaction between the exposure and percent MD

*Not adjusted for adolescent somatotype, BMI at age 18, or current BMI

^#^ Not adjusted for current BMI

NDE = Natural Direct Effect (i.e., the effect of the exposure on breast cancer risk *NOT through* percent density setting percent MD to the level in the unexposed)

NIE = Natural Indirect Effect (i.e., the effect of the exposure on breast cancer risk *through* percent density)

Percent of the total association between the exposure and breast cancer risk that was mediated by percent MD on the log odds scale was calculated using the formula 1-(lnOR_NDE_/lnOR_total_)

^Percent mediated calculated to be <0%

`^^Percent mediated calculated to be >100%
